# Application of artificial intelligence to eyewitness identification

**DOI:** 10.1186/s41235-024-00542-0

**Published:** 2024-04-03

**Authors:** Heather Kleider-Offutt, Beth Stevens, Laura Mickes, Stewart Boogert

**Affiliations:** 1 Department of Psychology, Georgia State University, Atlanta, GA 30030 USA; 2 School of Psychological Science, The University of Bristol, Beacon House, Queens Rd, Bristol, BS8 1QU UK; 3 Department of Physics and Astronomy, The University of Manchester, Oxford Rd, Manchester, M13 9PL UK

**Keywords:** Eyewitness accuracy, Face-recognition software, Estimator variables, Race effects, Viewing context, Legal implications

## Abstract

Artificial intelligence is already all around us, and its usage will only increase. Knowing its capabilities is critical. A facial recognition system (FRS) is a tool for law enforcement during suspect searches and when presenting photos to eyewitnesses for identification. However, there are no comparisons between eyewitness and FRS accuracy using video, so it is unknown whether FRS face matches are more accurate than eyewitness memory when identifying a perpetrator. Ours is the first application of artificial intelligence to an eyewitness experience, using a comparative psychology approach. As a first step to test system accuracy relative to eyewitness accuracy, participants and an open-source FRS (FaceNet) attempted perpetrator identification/match from lineup photos (target-present, target-absent) after exposure to real crime videos with varied clarity and perpetrator race. FRS used video probe images of each perpetrator to achieve similarity ratings for each corresponding lineup member. Using receiver operating characteristic analysis to measure discriminability, FRS performance was superior to eyewitness performance, regardless of video clarity or perpetrator race. Video clarity impacted participant performance, with the unclear videos yielding lower performance than the clear videos. Using confidence-accuracy characteristic analysis to measure reliability (i.e., the likelihood the identified suspect is the actual perpetrator), when the FRS identified faces with the highest similarity values, they were accurate. The results suggest FaceNet, or similarly performing systems, may supplement eyewitness memory for suspect searches and subsequent lineup construction and knowing the system’s strengths and weaknesses is critical.

## Introduction

The fallibility of eyewitness memory is well-documented in the scientific literature (Howe & Knott, 2015; Laney & Loftus, 2018; Pezdek, 2012), and eyewitness error has contributed to most cases being overturned by DNA evidence (Innocence Project, 2023), suggesting that memory errors that occur in the laboratory also occur in actual cases. Although imperfect, eyewitness identification may be the sole evidence in a criminal trial, influencing juror decisions (Albright & Garrett, 2022; Clark & Godfrey, 2009). As such, it is an integral part of the legal system, and understanding its reliability is paramount. Recent advances in artificial intelligence (AI) systems have led to the ubiquitous application of face recognition systems (FRS) expanding from the opening of a cell phone (i.e., face verification) to suspect searches (i.e., face identification) performed by police departments (Hill et al., 2022; Lynch, 2020). FRS is used primarily as a tool for law enforcement during the suspect identification process, but its performance is unclear, as much of the software is proprietary and search techniques vary. To date, no known study investigates whether FRS is a reliable tool for law enforcement such that the match returned as a potential suspect is on par or superior to eyewitness memory-based identifications. Akin to a comparative psychology approach, this study tests the discriminability and reliability of performance between FRS face-matching and human eyewitnesses face-match-to-memory.

How law enforcement uses FRS varies; they can pull a photo from security camera footage or social media and then run it through a database to search for a match, or a witness can provide a description or implicate familiar suspects whose identity is unknown, producing a photo, and using it as an image to search a database. Either way, the FRS returns potential matches, and the police decide which, if any, should be shown to the eyewitness (or other law enforcement) via an identification procedure. The question is whether FRS can produce possible matches that include the perpetrator better than an eyewitness can identify the perpetrator from recognition memory (i.e., using a match-to-memory process), if not, then FRS may not be a valuable tool for law enforcement or parameters around its use should be addressed.

Another question is if there are indicators of FRS accuracy—like confidence often is for identifications made from lineups (e.g., Wixted & Wells, 2017). Arguably, there are different approaches to compare eyewitness-FRS accuracy. Because this is exploratory, we chose a head-to-head comparison between human memory for perpetrators seen in a video (ID accuracy) and FRS's ability to use perpetrator pictures from the same video frames and provide matches from a pool of photos (by way of producing similarity scores). This is a similar approach taken by Richie et al. (2024), wherein performance of participants and AI algorithms were compared in a face matching task where the faces were partially occluded by masks. Like Richie et al., we employed an open-source FRS, FaceNet (Schroff, Kalenichenko, & Philbin, 2015), so that other researchers could replicate and further test this question with different stimuli. This does not mean that all systems will perform the same, i.e., due to variability in training, but this is the first attempt to run such a comparison (Adjabi et al., 2020; Firmansyah et al., 2023). We chose a system that is high performing, with some reports of accuracy rates being greater than 99% (Chaudhuri, 2020; Firmansyah et al. 2023). The reported accuracy rates only consider correct responses (Firmansyah et al. 2023), without regard to false alarms, and by presenting only correct responses gives an incomplete account of the systems’ true abilities (Government Accountability Office (GAO), 2016). We later suggest two types of analyses for measuring FRS performance that may better capture accuracy and errors that occur in real-world eyewitness contexts.

A variety of factors may influence eyewitness memory. Systems variables are controlled by the criminal justice system (e.g., lineup procedure and interview techniques; Wells, 1978). Estimator variables are inherent to the situation (e.g., distance, lighting, viewing brevity, and perpetrator race; Wells, 1978). During encoding, if the variables during the crime are impoverished, retrieval is negatively influenced (e.g., Giacona et al., 2021). For example, long viewing distances and low lighting result in lower discriminability, with both correct identifications decreasing and false identifications increasing (Lockamyeir et al., 2020; Nyman et al., 2019). One explanation is the strength-based Mirror Effect (Glanzer & Adams, 1985), which proposes that as the overall memory strength decreases, recognition performance decreases, as indicated by both decreased correct identifications and increased false identifications. This pattern occurs with poor lighting, long viewing distance (Davis & Peterson, 2022), and degraded video and lineup stimuli (DeJong et al., 2004; Smith et al., 2019; Wolters & Verstijnen, 2005).

The current work is especially relevant because law enforcement regularly uses surveillance footage, like closed circuit television (CCTV) programs. Ring camera surveillance is also used in investigations and made over 20,000 requests for home film footage in 2020 (Bridges, 2021; Harwell, 2021). Surveillance footage may vary in clarity, which, like long-distance viewing, may influence the strength of the initial event encoding. Whether the same difficulties befall an FRS when the stimuli are suboptimal is untested in an eyewitness paradigm.

Although relatively new, most people in the US view police officers' use of FRS and similar biometric systems as beneficial and trustworthy, especially when used in perpetrator identification (Rainie et al., 2022; Lynch, 2020; GAO, 2021). This acceptance likely comes from highly publicized and successful uses of FRS by law enforcement where the FRS match led to identifying people who were later charged with crimes (e.g., 2021 US Capitol insurrection perpetrator identifications; Cooper, 2021, and mass shooter John Ramos identification, Capital Gazette shooting; Parker, 2020). However, a different outcome occurred in another case, wherein FRS matched a Black man who was later released when the victim identified the true perpetrator from a lineup (i.e., Robert Williams; Cooper, 2021).

The National Institute of Standards and Technology (NIST), the only US governing body evaluating FRS algorithms (Facial Recognition Vendor Test; FRVT), found that algorithms produced higher false positive rates for People of Color than for White individuals when using law enforcement images (Grother et al., 2019). Data are mixed on whether there is a difference in failure to produce matches among different algorithms for these racial groups (Bowyer & King, 2019; Grother et al., 2019). FRVT also found that when image quality was poor, as with border crossing photographs, errors were highest, especially among Black people (Grother et al., 2019). However, better algorithms have improved accuracy rates in recent years (Hanacek, 2018).

Although there are concerns about FRS accuracy with poor quality input (e.g., Golla & Sharma, 2019), as with impoverished viewing conditions for eyewitnesses, these factors that influence perception, and thus potentially compromise the original memory, can impact recognition accuracy (e.g., Nyman et al., 2019; Wixted et al., 2018a, 2018b). While discriminability may be affected, reliability may not be (e.g., Mickes, 2015; Semmler et al., 2018). Suggesting that, like confidence is an indicator of accuracy for witnesses, the strength of the similarity score returned from the FRS may also be indicative of accuracy. This information may be useful when determining whether a match or identification is useful evidence.

Mickes (2015) made a distinction between two types of eyewitness identification accuracy. The first type of accuracy is discriminability (i.e., distinguishing innocent from guilty suspects). Discriminability is measured using receiver operating characteristic (ROC) analysis, which involves plotting correct ID and false ID rates for every level of confidence. Researchers often refer to this kind of accuracy when discussing results from identification experiments. However, there is another type of accuracy, referred to as "reliability." Mickes introduced confidence-accuracy characteristic (CAC) analysis, which involves computing proportion correct for different confidence levels to measure reliability. Thus, CAC analysis provides information about the relationship between confidence and suspect identification accuracy. Results from these analyses led many researchers to believe confidence is informative of accuracy (e.g., Wixted & Wells, 2017; Seale-Carlisle et al., 2024). As with human performance on identification procedures, both types of accuracy are useful in assessing FRS performance, as we do here.

Amid the uncertainty surrounding FRS and their improving accuracy rates, questions are raised regarding FRS's superiority to human eyewitnesses' performance. Recent studies investigating FRS and human performance have typically found combining both yields the best outcome, but these studies use still-front-facing photos of White or Asian "perpetrators" (e.g., Phillips, 2018; White et al., 2015). To test the limits of FRS and how it compares to humans, in the current study, we presented the FRS and participants, crime videos taken from actual surveillance footage, wherein the perpetrator positioning and video quality varied. While the human participants probed their memories for the perpetrator, the FRS identified the face of the perpetrator in each frame of the video and then provided a similarity score (to the perpetrator) for each face in the lineups. This procedure makes finding a match more challenging than comparing clear static pictures with database pictures. In addition, as racial bias may be inherent in FRS systems, here, Black, Hispanic, and White perpetrators were in the videos.

We compared the discriminability of FRS and human participants via ROC curves and the reliability via CAC curves. This study is exploratory for FRS performance; however, for the human data, we anticipated outcomes to align with previous studies such that poor clarity will decrease discriminability. We expect identifications made with high confidence will be more accurate than lower confidence identifications. Given the racial diversity of our participant population (37% Black, 30% White, 11% Hispanic/Latinx, etc.), we did not expect perpetrator race to influence accuracy.

## Methods

### Participants

Participants (*N* = 237) were recruited from Georgia State University's undergraduate subject pool and *Prolific* (www.prolific.com) [May 2023]. GSU's IRB approved the protocol. All participants gave consent before participating. Participants were 18 to 66 years old, and the majority identified as Black (*n* = 88; 72 White; 27 Asian; 29 Hispanic/Latinx; 8 Bi-racial, and 13 as other); female (64.98%; *n* = 154), male (32.07%, *n* = 76), and non-binary or no answer (2.95%, *n* = 7).

### Materials

#### Video stimuli

**Fig. 1:**
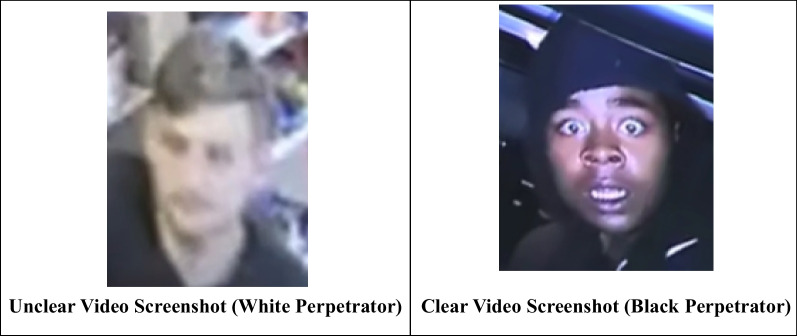
Screenshots from the clear and unclear videos. There were 3 clear and 3 unclear videos (one for each perpetrator race)

Pre-ratings for all video stimuli were obtained from 40 participants (majority Black; *n* = 15, 37.5%), female (*n* = 32, 80%), and majority aged from 18 to 23 (*n* = 39, 97.5%). Six videos featured 2 White, 2 Black, and 2 Hispanic perpetrators. For each race/video condition, there was a higher quality video (i.e., clear video) and a lower quality video (i.e., unclear video). See Fig. 1 for clear and unclear video screenshots. Video selection was based on the perpetrators’ race and the videos' clarity ratings, determined by those with the lowest and highest clarity ratings. The average clarity rating for the lower-quality videos ranged from 51.15 to 60.50 (100-point scale), while the average clarity rating for the higher-quality videos ranged from 71.65 to 84.03. All crime scene videos depicted either a robbery, break-in, or purse snatching, and the perpetrator's face was in view for an average of 8.5 s across all videos (taken from news sources ABC7, 2019; CBS Fox 59, 2020; Fort Worth Star-Telegram, 2020; Officers capture robbery suspect who used electric weapon, n.d.; WESH2, 2019).

#### Facial stimuli

Images of the perpetrators used in the lineups were taken from police report photographs of the perpetrator associated with the arrest of the depicted crime (i.e., a mugshot). Filler faces were from the Chicago Face Database (Ma et al., 2015) and the State of Florida's website of mugshots (Arrests.org, 2023). For each video, 6 filler images were edited to ensure comparable image quality. The filler faces provided for both the target-absent and target-present lineups were pre-rated by either 20 participants in a previous study (Kleider et al., 2021) or by 44 additional participants for the current study, the majority Black (*n* = 17; 38.6%), female (*n* = 25; 56.8%), and aged from 18 to 23 (*n* = 42; 95.5%).

Faces in both pre-rating studies were rated on how similar the face was to the actual perpetrator on the following attributes: attractiveness, looks, age, appearance, and facial features. The similarity ratings were recorded on a scale of 0 (*not at all similar*) to 100 (*completely similar*), and the five similarity attribute ratings were averaged for each face. The faces with the highest similarity ratings were selected as the high-similarity innocent suspects in the target-absent lineups (to replace the perpetrator in target-present lineups). The remaining filler faces comprised the next highest similarity ratings among the pool of faces. Average similarity ratings across the faces ranged from approximately 17.00 to 35.00, comparable to similarity ratings used for stimuli in other studies (Kleider-Offutt et al., 2021). Filler faces selected for the lineup followed the suggestion that filler faces for a lineup should be selected based upon a match to description of similarity to the perpetrator, but with caution to avoid too much similarity to the perpetrator (e.g., Lucas et al., 2021; Wells et al., 1993; Wooten et al., 2020)).

#### Lineups

Memory was tested on 6-person simultaneous lineups in 2 x 3 arrays—the target-present lineups comprised images of the perpetrator's face and five filler faces. In the target-absent lineups, high-similarity innocent suspects replaced the perpetrators. The image orders were randomly placed per participant.

#### Facial recognition system technology

Google’s open-source FRS, FaceNet (Schroff et al., 2015), was chosen given its accessibility to the public. It is also used with highly trained algorithms for face detection and alignment, and recognition. (From this point on, references to FRS specifically refer to FaceNet). Chaudhuri (2020) and Firmansyah et al. (2023) reported that FaceNet yielded high accuracy rates, as high as 99.63% and 99.2%, respectively, when tested on the faces in the Labeled Faces in the Wild database (Huang et al., 2007). Note, again, these rates only include correct responses, not incorrect responses (Firmansyah et al. 2023).

For face detection, the Multi-Task Cascaded Convolutional Neural Network (MTCNN; Dulcic, 2020; Zhang et al., 2016) was used. For facial recognition, a pre-trained model, InceptionResnetV1, was used (Esler, 2023). It is an algorithm trained against the VGGFace2 database of 3.3 million images, akin to those used by the Department of Defense (see Cao et al., 2018). For a review of FRS open-source system performance see Adjabi et al., 2020; Firmansyah et al., 2023.

### Design and procedure

The current study consisted of two procedural elements. First, human participants participated in an online eyewitness identification task via Qualtrics. The second element involved a facial recognition task by the open-source FRS described above.

#### Online human participants

**Fig. 2:**
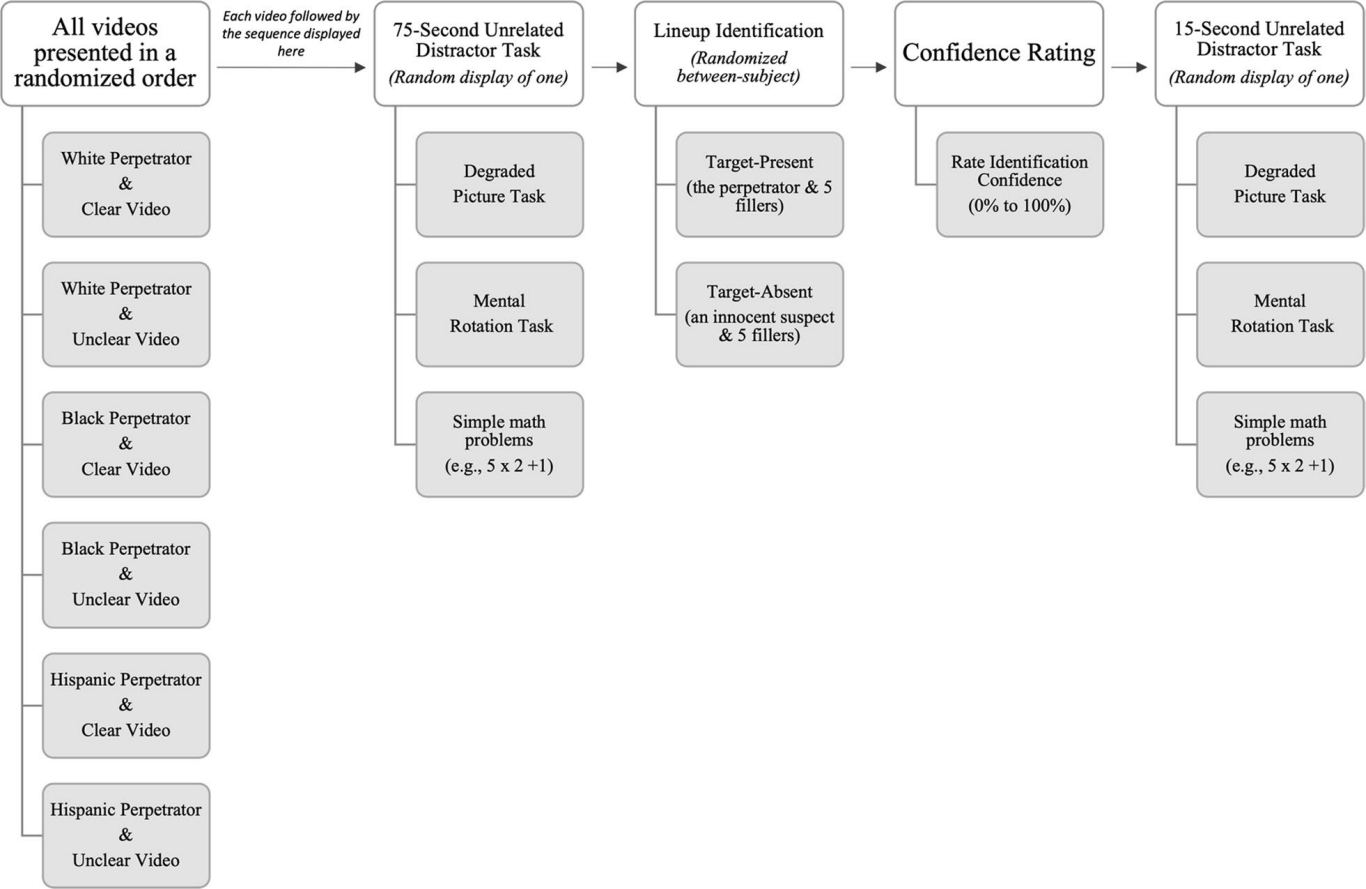
Diagram of the procedure for the human participants. Participants viewed all six videos in a random order. After viewing each video, participants completed the same sequence of events (distraction task one, identification task of a target-present or target-absent lineup, confidence rating, distractor task two). Prior to each identification task, participants read standard instructions

See Fig. 2 for an illustration of the procedure. Participants viewed all six videos. As is standard procedure in lineup experiments, following the viewing of each video, participants completed a distraction task (e.g., degraded picture task, mental rotation task) of approximately 75 s in length before completing a corresponding identification task to mimic delays and competing information that may influence encoding processes. During the identification task, participants tried to identify the perpetrator shown in the videos from either target-absent or target-present lineups or selected the "not present" option and rated their confidence on a scale ranging from 0 (*not at all confident)* to 100 (*completely confident*). After completing the identification task, participants viewed another distraction task lasting approximately 15 s before completing the sequence again for each of the videos. This second distractor task was implemented to reduce source memory/monitoring issues between the previously viewed lineup and the next video/lineup sequence. The order in which each participant completed this sequence was randomized by video to control for order effects. Participants also reported their age, gender, and race. The entire study took approximately 40 to 60 min to complete.

#### Facial recognition system procedure

For the FRS element, the same six videos and corresponding images (those that made up the target-present and target-absent lineups) that were presented to participants were used. As standard practice in FRS use, the FRS extracted a frame of each perpetrator's face^1^ to create a probe of the perpetrator through the face detection process. It is difficult to compare the accuracy of the FRS with human performance using a single probe image, and it is not conducive to conducting ROC and CAC analyses. However, this is the process law enforcement uses when conducting an FRS search. Thus, we followed the same procedure. We compared each probe image from each video with each of the lineup images. Euclidean distances (similarity scores) were produced for each probe-lineup image pair, with the lowest values indicating the strongest similarities (see Table 3 in Appendix 1).

## Results

The data and code are available at OSF (https://osf.io/6tfuj/?view_only=c0ea0e5d02b34a529e1366f8daac62da). The analyses were conducted in pyWitness (https://lmickes.github.io/pyWitness/; Mickes et al., 2023).

### FRS results

All frames in the videos can be compared to the lineup images using the FRS. This comparison introduces significant variation in face orientation, lighting, distance, and size of the face in the image, which explores the response of the FRS to the entire video. We plotted histograms showing the similarity values for guilty suspects, innocent suspects, and fillers. Performance varied across videos, as shown in Fig. 7 in Appendix 2.

Correct, false, and filler IDs were computed for the data generated by participants (Table 4 in Appendix 3). We binned the confidence responses of the participants and Euclidean values of the FRS into 6 bins each so that each bin had similar numbers of responses. Confidence responses were collapsed into six bins: [0–20], (20–40], (40–60], (60–80], (80–90], (90–100]. For the FRS data, correct IDs are the number of guilty suspects with the closest similarities for perpetrators per video frame. The false IDs are the number of innocent suspects with the closest similarities per video frame. The Euclidean values were binned into 6 categories: [0.7–1.1], (1.1–1.2], (1.2–1.25], (1.25–1.3], (1.3–1.35], (1.35–1.6]. Low values indicate higher similarities.^2^

### Overall discriminability

To compare the discriminability of the participants and FRS, we conducted confidence-based (e.g., Grounlund et al., 2014) and similarity value-based ROC analyses, respectively. A ROC plots correct ID rate and false ID rate pairs for every level of confidence or similarity, cumulating as confidence decreases or similarity increases (e.g., Gronlund et al., 2014; Mickes et al., 2023). The further the points bow toward the upper left corner, the better the ability to discriminate innocent from guilty suspects. To statistically compare ROC curves in lineup data, partial area under the curve (pAUC) values are computed. To compute pAUC values, a false ID cut-off must first be determined. The standard practice is to choose a false ID cut-off, often from the condition that yields the lowest maximum false ID rate. We used 95% confidence intervals to make the statistical inferences (e.g., on pAUC comparisons). We used 68% confidence intervals on the plots because given the correlations in the bootstrap samples, it is likely an overestimate of the variability.

**Fig. 3:**
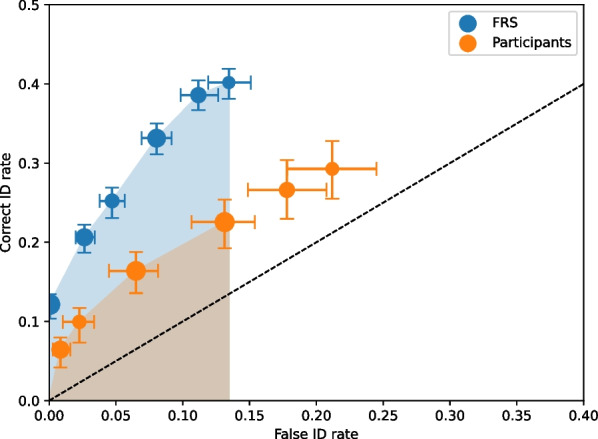
Receiver operating characteristic (ROC) curves for the face recognition system (FRS) and participants. The shaded regions represent the partial area under the curve (pAUC) regions for each curve, using the cut-off of the overall false ID rate of the FRS. The error bars are 68% confidence intervals based on 200 bootstraps. The dashed line represents chance performance. Point sizes reflect relative frequencies of responses

Figure 3 shows the ROC curves for the participants and FRS collapsed across videos and conditions (video clarity and perpetrator race). Table 1 shows the pAUC values and false ID cut-off. The FRS had a significantly higher pAUC value than the participants, *Z* = 3.2577, *p* = 0.0011. Thus, the FRS could significantly better discriminate between guilty and innocent suspects.

**Table 1:** False ID cut-offs, pAUC values, and standard errors for the FRS and participants collapsed across conditions and for each condition

	False ID cut-off	pAUC	± se
*Collapsed across condition*			
FRS	0.1344	0.0718	0.0155
Participants		0.0210	0.0021
*Clarity condition*			
FRS			
Clear	0.1114	0.0609	0.0135
Unclear		0.0269	0.0016
Participants			
Clear		0.0220	0.0030
Unclear		0.0118	0.0021
*Race condition*			
FRS			
Black	0.0842	0.0309	0.0063
Hispanic		0.0091	0.0013
White		0.0195	0.0018
Participants			
Black		0.0120	0.0024
Hispanic		0.0090	0.0017
White		0.0147	0.0033

### Discriminability comparisons by conditions

#### Clarity conditions

Collapsing across perpetrator race, we compared discriminability from the clear versus unclear conditions. The FRS clear videos yielded the lowest overall false ID rate, and this is the cut-off we used to compute pAUC values for the clear and unclear videos. We made four pAUC comparisons: FRS clear versus FRS unclear, participants clear versus participants unclear, FRS clear versus participants clear, and FRS unclear versus participants unclear. To correct for multiple comparisons, we used Bonferroni correction of 0.0125 (0.05/4) for the significance threshold value.

**Fig. 4:**
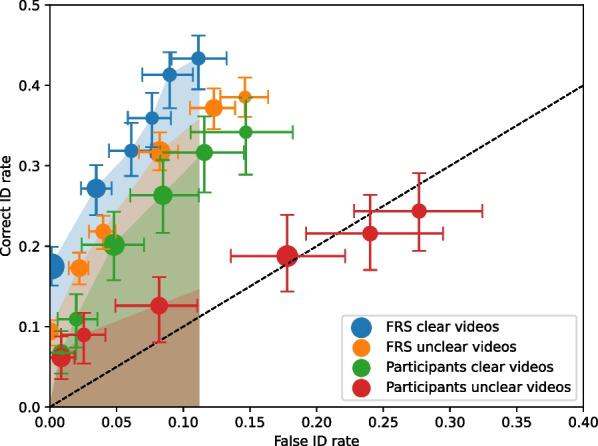
Receiver operating characteristic (ROC) curves of the FRS and participants for the clear and unclear conditions. The shaded regions represent the partial area under the curve (pAUC) regions for each condition, using the cut-off of the overall false ID rate of the FRS clear condition. The error bars are 68% confidence intervals based on 200 bootstraps. The black dashed line represents chance performance. Point sizes reflect relative frequencies of responses

The pAUC values are presented in Table 2 (along with the false ID cut-off), and the ROC curves are shown in Fig. 4. The FRS performance with clear videos was higher than its performance with unclear videos, but the difference was not significant after the Bonferroni correction, *Z* = 2.4941, *p* = 0.0126, but was close. Participant performance with clear videos was significantly higher than their performance with unclear videos, *Z* = 2.7775, *p* = 0.0055. The FRS performance with clear videos was significantly higher than the participants' performance with clear videos, *Z* = 2.8001, *p* = 0.0051. The FRS performance with unclear videos was also significantly higher than the participants' performance with unclear videos, *Z* = 5.8147 *p* < 0.0001.

**Table 2:** *Z*-values and *p*-values for the race comparisons

Comparisons	*Z*-value	*p*-value
FRS Black versus FRS White	1.7451	0.0810
FRS Black versus FRS Hispanic	3.3991	0.0007*
FRS Hispanic versus FRS White	4.7014	< 0.0001*
Participant Black versus Participant White	0.6541	0.5130
Participant Black versus Participant Hispanic	0.0256	0.3050
Participant Hispanic versus Participant White	1.5393	0.1237
FRS Black versus Participant Black	2.8052	0.0050*
FRS Hispanic versus Participant Hispanic	0.0675	0.9462
FRS White versus Participant White	1.2693	0.2043

*Significant differences after the Bonferroni corrections

### Race conditions

**Fig. 5:**
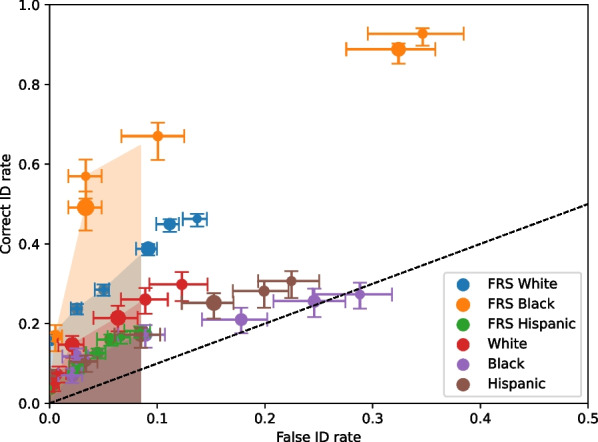
Receiver operating characteristic curves for the race conditions. The shaded regions represent the pAUC per condition, using the cut-off of the overall false ID rate of the FRS White condition. The error bars are 68% confidence intervals based on 200 bootstraps. The dashed line represents chance performance. Point sizes reflect relative frequencies of responses

Collapsing across the clarity conditions, we compared discriminability of the FRS and participants from the videos featuring Black, Hispanic, and White perpetrators. Using Bonferroni corrections for multiple comparisons (*p* = 0.05/9 = 0.0056), there were three significant differences between groups, as shown in ROC curves in Fig. 5 and Table 2. The significant differences were in the FRS comparisons of the Black versus Hispanic pAUCs, Hispanic versus White pAUCs, and the FRS comparison with participants with the Black videos.

### Confidence accuracy characteristic analysis

**Fig. 6:**
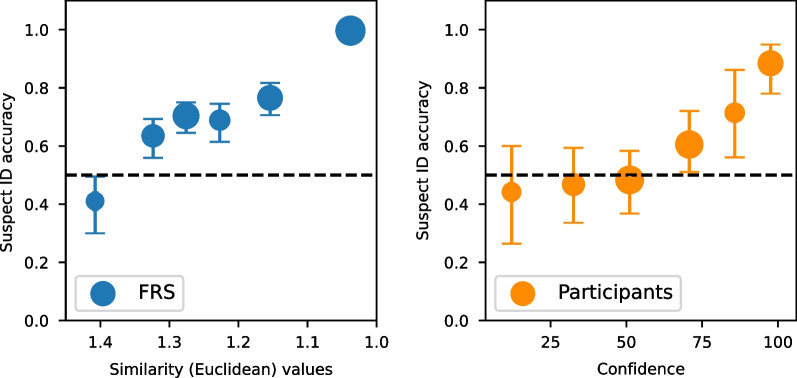
Confidence accuracy characteristic plots collapsed across conditions for the FRS (left panel) and participants (right panel). The error bars are 68% confidence intervals based on 200 bootstraps. The black dashed line represents chance performance. Point sizes reflect relative frequencies of responses. The faces with the strongest FRS similarity values were 100% accurate (< 1.1)

**Fig. 7:**
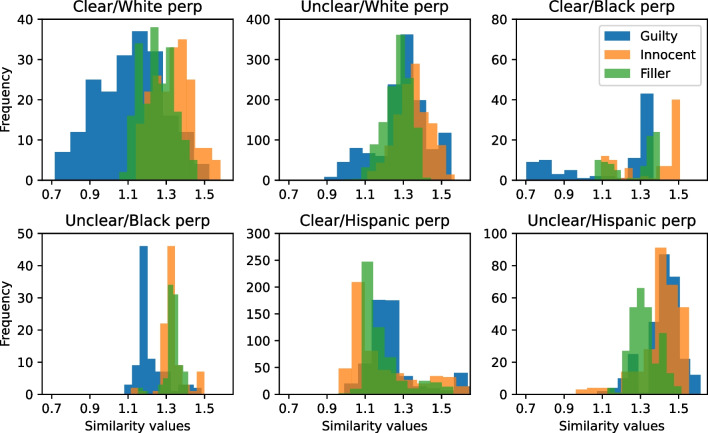
Histograms of the Euclidean similarity values for guilty suspect, innocent suspect, and filler distributions for each video. Lower values indicate higher similarities. Values on the y-axis vary as a result of the different number of frames in each video

**Fig. 8:**
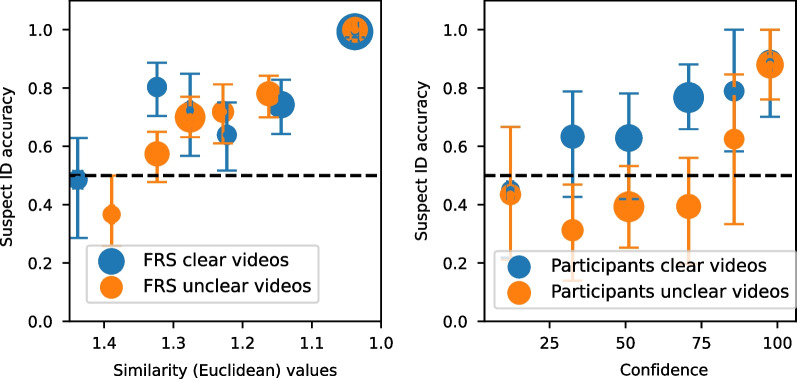
Confidence accuracy characteristic plots collapsed across race for the FRS (left panel) and participants (right panel). The error bars are 68% confidence intervals based on 200 bootstraps. The black dashed line represents chance performance. Point sizes reflect relative frequencies of responses

**Fig. 9:**
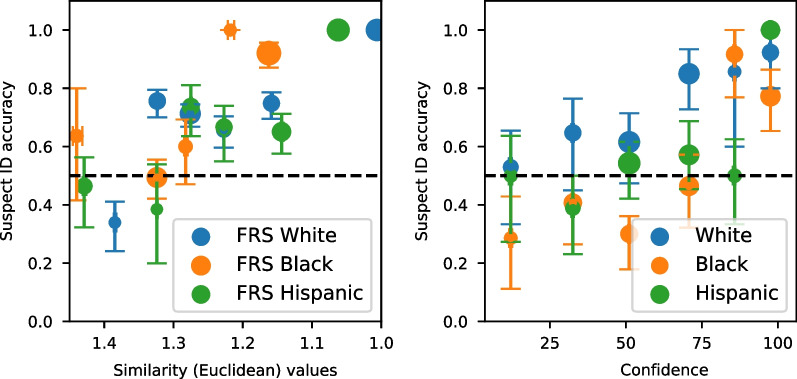
Confidence accuracy characteristic plots collapsed across clarity for the FRS (left panel) and participants (right panel). The error bars are 68% confidence intervals based on 200 bootstraps. The black dashed line represents chance performance. Point sizes reflect relative frequencies of responses. The faces with the strongest FRS similarity values were 100% accurate (< 1.1)

CAC analysis, a graphical analysis, involves plotting suspect ID accuracy (#correct suspect ID/(#correct suspect IDs + #incorrect suspect IDs)) for each level of confidence (for the participants) and similarity values (for the FRS). The left panel in Fig. 6 shows the FRS CAC plot, and the right panel shows the participants' CAC plot. Identifications made with higher confidence were generally higher in accuracy than lower identifications made with lower confidence. For the strongest similarity values, FRS performance was perfect (i.e., suspect ID accuracy of 1.0). The CAC plots broken down by clarity and race are in Figs. 8 and 9 in Appendix 4.

## Discussion

We tested an open-source FRS (FaceNet with MTCNN & VGG2) on 6 videos that vary on multiple factors, including clarity and perpetrator race. Both factors have, in the past, impacted FRS abilities (Adjabi et al., 2020; Grother et al., 2018). We also tested human performance on lineups after witnessing the same six videos. This paper is the first to use ROC and CAC analyses with FRS data from videos of real crimes, allowing us to assess discriminability and reliability, respectively.

Focusing on the FRS performance, we observed that discriminability was not perfect (i.e., indicating some overlap between the distributions of innocent and guilty suspects). However, the FRS had better discriminability than the human participants. This result is unsurprising given the task was more challenging for humans, who relied on recognition memory (often considered as match-to-memory process), while the FRS matched stimuli. In other words, the humans were disadvantaged because the study stimuli were not available to interrogate during the test phase, they used their memory to match the test stimuli, whereas the FRS jointly interrogated the study and test stimuli. While the processes are likely not analogous, FRS and eyewitness performance showed similar trends to race and video quality. Lower-quality videos led to smaller pAUCs for both the FRS and participants.

It is important to highlight that we do not claim the FRS and participants use the same processes to complete the task, nor do we claim to have gained new insight into human memory based on the FRS results. While comparisons between AI and human performance have been conducted where both complete a matching task (e.g., Ritchie et al., 2024), it is unlikely that they use the same processes to complete the task. Ritchie et al., made this point, stating,

It is worth noting that we do not suggest that human observers and algorithms are equivalent or are performing the task in the same way. (p. 10)

The comparison between FRS and human performance, as demonstrated by Ritchie et al. (2024), provides context regarding the usefulness of FRS—aligning with one of our study’s objectives. We employed a comparative psychology approach to understand human-AI capacities, similar to studies attempting to link animal cognition to AI to understand shortfalls of either system (Crosby et al., 2019). Here we aimed to understand FRS and human performance on a lineup task, and in doing so, we tried to avoid potential biases (such as anthropomorphism; Buckner, 2019). This involved avoiding the erroneous assumption that FRS and humans use identical processes or mechanisms to accomplish the same task. In this sense, our study is unlike conventional lineup studies, where groups of participants are typically compared under different conditions, leading to interpretations about underlying processes. We believe that limiting the use of FRS solely to matching tasks would be overly restrictive, given the myriad potential applications of FRS, including some we have previously mentioned (and probably many more we have yet to consider). Understanding the capabilities of AI, including in the context of varied video stimuli, was a key focus of our investigation. We believe this broader understanding is essential for maximizing the potential of FRS in various scenarios.

As found in previous studies with impoverished stimuli (Golla & Sharma, 2019; Smith et al., 2019), the FRS overall correct ID rate was reduced for the unclear (39%) versus clear (43%) video and the overall false ID rate was increased for the unclear (15%) versus clear (11%) video. The same pattern arose with the participant data, where the overall correct ID was reduced for the unclear (24%) versus clear (34%) video and the overall false ID rate was increased for the unclear (28%) versus clear (15%) video. This pattern represents the Mirror Effect (Glanzer & Adams, 1985). Memory strength influences the ability of a witness to accurately recognize the perpetrator's face as the perceptual details were unavailable at encoding, when an identification was made, the correct ID rate was low and the false ID rate was high relative to a clear video as expected.

Another factor that affects identification performance, especially with surveillance video, is viewing the perpetrator's face from different angles rather than straight on, which is inconsistent with lineup presentation (Colloff et al., 2020). If an interactive lineup (Colloff et al., 2022; Meyer et al., 2023) were used, it is possible discriminability would be higher as participants could move the lineup faces to be in a similar orientation at retrieval that they viewed at encoding to take advantage of encoding specificity (i.e., encoding specificity principle; Tulving & Thomson, 1973). Although these challenges are part of a real-world identification scenario, the mismatch between face presentation between encoding (viewed from the side or above) and retrieval (lineup) may have contributed to the overall low discriminability for eyewitnesses.

FRS had similar difficulty matching a perpetrator's face when clarity was suboptimal but to a lesser extent than eyewitnesses, suggesting that even high performance FRS have limitations when stimuli are suboptimal. News reports about inconsistent FRS performance across different races raised ongoing concerns about whether the systems are biased. In the current study, the FRS system generally performed better than the participants regardless of perpetrator race, except for the Hispanic videos, suggesting that the FRS used here did not produce biased output. In addition, although overall performance was better for the FRS, the eyewitness data did not show differences by perpetrator race, which is likely due to the participant diversity. This finding may also be partly due to the improved training of current algorithms tested by NIST, which has seen significant improvements in submitted algorithms' accuracy rates with less bias in recent years (Grother, 2021; Hanacek, 2018). However, that cannot account for all FRS. In addition, although the video stimuli were pre-rated, in the video with a Black perpetrator, his face was especially prominent, which may also have contributed to the similar performance across perpetrator race. A study wherein video quality is maintained and controlled while only manipulating perpetrator race could address this question.

The FRS discriminability was superior to eyewitness identifications regardless of video clarity. Both FRS and eyewitness performance dropped when viewing conditions were impoverished, although not significantly when comparing FRS clear versus unclear but discriminability was significantly higher for participants with clear versus unclear videos. Although FRS performance was superior to participants, for one Hispanic video (Fig. 5), the FRS yielded a small area under the ROC curve, which speaks to the limitation of FRS systems (Adiabi et al., 2020).

Unlike ROC analysis, which provides information about the ability to discriminate between guilty and innocent suspects, CAC analysis provides information about suspect ID accuracy at a given level of confidence (Mickes, 2015) or a given level of similarity value, as in the FRS case. Participants' high-confidence responses were higher in accuracy than lower-confidence responses. The pattern of results is in line with CACs of other studies (e.g., Wixted & Wells, 2017), showing that participants have metacognitive awareness. That is, they use confidence to indicate their likelihood of making an error (Mickes et al., 2012). If the chance of making an error is low, an identification will be made with high confidence. And if it is high, an identification will be made with low confidence.

Remarkably, we observed a consistent pattern in the FRS CAC results, wherein the highest similarity values showed higher accuracy compared to weaker similarity values. Notably, the strongest similarity values exhibited perfect accuracy of 100% (Fig. 6, left panel). Should this pattern be consistently replicated across various settings and testing different systems, it would suggest that high-similarity matches could be reliable. Considering these findings, and future replications, it may be sensible for law enforcement to consider prioritizing images with strong similarity values to minimize the risk of including innocent suspects in lineups.

The use of FRS as a tool for law enforcement lacks comprehensive testing, and there are currently no regulations or standardized officer training programs for interpreting outcomes (Lynch, 2020), although government agencies are aware training is needed (GAO, 2023). Our findings suggest that the FRS we used, FaceNet, hold promise as a valuable asset for law enforcement agencies. It is crucial to understand its capabilities, particularly when dealing with low-quality stimuli, which are often the only resources available to law enforcement personnel. While our study revealed that discrimination performance was not error-free, it is worth noting that the highest similarity values were highly accurate. Thus, output (e.g., match) interpretation could be key.

While this study represents initial exploration in this area, there remains extensive future work to be done. This includes systematically varying the factors in the videos, employing different types of stimuli, and exploring different AI systems. We recommend the use of ROC and CAC analyses to provide insights into accuracy for future FRS investigations.

### Practical implications

According to the Washington Post (Harwell, 2022), Clearview AI was projected to have the face of nearly everyone worldwide in their database by the end of 2022 (their website claims to have 30 billion face images, https://www.clearview.ai/post/how-we-store-and-search-30-billion-faces, retrieved February 23, 2024). This projection suggests that for FRS database searches for a perpetrator, there is no "target-absent" search, as everyone is, or will be, in the database. With FRS currently used by law enforcement during the initial investigation to generate potential leads or suspects, defendants may face severe limitations in contesting the use of FRS for identification purposes. Ultimately, a police officer makes the final decision regarding the utility of the FRS match, leaving room for human error. To reduce potential errors, our data indicate that officers may benefit from considering the similarity values of the FRS system. However, this recommendation should be considered alongside other corroborating evidence suggesting *reasonable suspicion,* following established best practices guidelines (Wells et al., 2020).

### Limitations

Only one FRS system was tested, and other systems with different similarity ratings may produce different results. In addition, we did not investigate cross-race effects in this study. Future research with larger samples of different racial groups may test whether participant differences influence performance. This study is a first attempt at comparing eyewitnesses and FRS in a controlled setting, and given that FRS is becoming ubiquitous in everything from cell phone access to airport security to CCTV to ring cameras to social media, other studies should be conducted to test FRS in different contexts.

## Conclusions

Law enforcement often relies on eyewitness identifications to corroborate FRS matches and produce admissible eyewitness identifications, potentially leading eyewitness to believe that the FRS-generated suspect is indeed the perpetrator. While our study revealed that the particular FaceNet FRS we used outperformed eyewitnesses even when the video quality was poor, its discrimination performance was not perfect. Our study also revealed that the faces with the strongest similarity values were accurate, potentially providing some useful guidance for practitioners. Before making these kinds of recommendations, multiple replication studies, across various systems (including the proprietary systems used by law enforcement agencies) using a large number of different stimuli, should be conducted. Without consistent guidelines, this finding could vary as a function of the system used. The potential for FRS to act in concert with eyewitness identification has the potential to reduce misidentifications, provided the FRS is properly vetted and legal guidelines are created to determine what is considered admissible evidence.

## Data Availability

The data and scripts are available at OSF (https://osf.io/6tfuj/?view_only=c0ea0e5d02b34a529e1366f8daac62da). The analyses were conducted in pyWitness (https://lmickes.github.io/pyWitness/; Mickes et al., 2023).

## Appendix 1: Comparisons of FRS single probe image to the line up images

Table 3 shows the probe image similarity values to images of the guilty suspects, innocent suspects, and fillers. In five out of the six videos (videos 1–4, 6), the probe images were most similar to the guilty suspect images. In video 5 (clear-Hispanic), the probe image was most similar to a filler image. The FRS performed well, but not perfectly.

**Table 3:** Probe/perpetrator similarity Euclidean values to the lineup images in each video

	Clear/White	Unclear/White	Clear/Black	Unclear/Black	Clear/Hispanic	Video 6 unclear/Hispanic
Probe versus guilty suspect	**0.8759**	**1.0740**	**0.8387**	**1.0122**	1.2415	**1.1231**
Probe versus image 2	1.2342	1.2859	1.0387	1.2232	1.4467	1.2652*
Probe versus image 3	1.3474	1.3318	1.1547	1.2503*	**1.1473**	1.2791
Probe versus image 4	1.2398*	1.1821*	1.2915	1.4450	1.3645*	1.4459
Probe versus image 5	1.2717	1.3981	1.1154*	1.2667	1.1586	1.4535
Probe versus image 6	1.2998	1.2208	1.1932	1.4921	1.4552	1.2728
Probe versus image 7	1.2906	1.2254	1.1042	1.3032	1.2347	1.2026

Bolded values show the strongest similarities

*Designated innocent suspect

## Appendix 2: FRS similarity histograms of suspect IDs and filler IDs

To create the histograms, when the FRS detected a face in each frame from each video, similarity scores were produced between all the lineup images and the images in the frames (this accounts for viewing variation). Figure 7 shows the similarity value for all video frames compared to guilty, innocent and filler lineup images in a histogram. To interpret these histograms, a high-performing FRS should have a distribution of video frame and guilty similarity that is furthest to the left, i.e., the smallest Euclidean distance. The FRS performance is perfect if there is no overlap between guilty and fillers. If there is complete overlap, then the FRS is effectively making a random choice. This can be thought of as a signal detection-like interpretation.^3^ The number of entries in each histogram is proportional to the number of frames in the video and has no bearing on the interpretation of the histograms. There is variation in the FRS performance, where some histograms showed much overlap between the distributions of similarity scores for the guilty suspects, innocent suspects, and fillers, and in some, more apparent separations arose. Also, some videos yielded stronger similarity for the fillers than for the guilty suspects (Hispanic clear and unclear videos) and some yielded higher similarity scores for the guilty suspects than the other distributions (all other videos). One interesting finding arose where the innocent suspect distributions were shifted to the right of the filler distributions (except for the clear and unclear Hispanic videos). What humans deem most similar may not be the same for the FRS.

## Appendix 3: Participant data

For the data generated by participants, correct IDs are identifications of the perpetrators (guilty suspects) in the target-present trials. Correct ID rates are computed by dividing the number of perpetrators correctly identified by the total number of target-present lineups. False IDs are identifications of the innocent suspects in the target-absent lineups. False ID rates are computed by dividing the number of innocent suspects incorrectly identified by the total number of target-absent lineups. Filler IDs are identifications of fillers from target-present or target-absent lineups. Table 4 shows the number of correct IDs, false IDs, filler IDs, and reject IDs (participants who selected the "not present" option) by level of confidence. Confidence responses were collapsed into six bins: [0–20], (20–40], (40–60], (60–80], (80–90], (90–100].

**Table 4:** Participants' number of correct IDs, false IDs, filler IDs, and reject IDs by level of binned confidence for target-present and target-absent lineups collapsed across clarity and race conditions

Lineup	Response type	Confidence					
		0–20	21–40	41–60	61–80	81–90	91–100
Target-absent	Innocent suspect ID	24	33	47	30	10	6
	Filler ID	54	65	119	93	32	29
	Reject ID	16	29	42	36	16	27
Target-present	Filler ID	82	59	71	62	25	27
	Reject ID	34	18	47	38	15	27
	Suspect ID	19	29	44	46	25	46

## Appendix 4

Figures 8 and 9 are the FRS and human participants’ CAC plots for clarity (collapsed across race) and race (collapsed across clarity).

## Footnotes

1 MTCNN provides a confidence value for each image and the probe with the highest value was selected from all frames of the video.

2 The FRS always returns a value; it does not have a reject ID response, although a similarity threshold could be used.
